# The START Study to evaluate the effectiveness of a combination intervention package to enhance antiretroviral therapy uptake and retention during TB treatment among TB/HIV patients in Lesotho: rationale and design of a mixed-methods, cluster-randomized trial

**DOI:** 10.3402/gha.v9.31543

**Published:** 2016-06-27

**Authors:** Andrea A. Howard, Yael Hirsch-Moverman, Koen Frederix, Amrita Daftary, Suzue Saito, Tal Gross, Yingfeng Wu, Llang Bridget Maama

**Affiliations:** 1 ICAP, Columbia University, New York, NY, USA; 2 Department of Epidemiology, Columbia University, New York, NY, USA; 3 CAPRISA, Nelson R. Mandela School of Medicine, University of KwaZulu Natal, Durban, South Africa; 4 Dalla Lana School of Public Health, University of Toronto, Toronto, Canada; 5 Department of Health Policy and Management, Columbia University, New York, NY, USA; 6 Lesotho Ministry of Health National Tuberculosis Program, Maseru, Lesotho

**Keywords:** TB/HIV integration, implementation science, cost-effectiveness, acceptability, TB treatment success

## Abstract

**Background:**

Initiating antiretroviral therapy (ART) early during tuberculosis (TB) treatment increases survival; however, implementation is suboptimal. Implementation science studies are needed to identify interventions to address this evidence-to-program gap.

**Objective:**

The Start TB Patients on ART and Retain on Treatment (START) Study is a mixed-methods, cluster-randomized trial aimed at evaluating the effectiveness, cost-effectiveness, and acceptability of a combination intervention package (CIP) to improve early ART initiation, retention, and TB treatment success among TB/HIV patients in Berea District, Lesotho.

**Design:**

Twelve health facilities were randomized to receive the CIP or standard of care after stratification by facility type (hospital or health center). The CIP includes nurse training and mentorship, using a clinical algorithm; transport reimbursement and health education by village health workers (VHW) for patients and treatment supporters; and adherence support using text messaging and VHW. Routine data were abstracted for all newly registered TB/HIV patients; anticipated sample size was 1,200 individuals. A measurement cohort of TB/HIV patients initiating ART was recruited; the target enrollment was 384 individuals, each to be followed for the duration of TB treatment (6–9 months). Inclusion criteria were HIV-infected; on TB treatment; initiated ART within 2 months of TB treatment initiation; age ≥18; English- or Sesotho-speaking; and capable of informed consent. The exclusion criterion was multidrug-resistant TB. Three groups of key informants were recruited from intervention clinics: early ART initiators; non/late ART initiators; and health care workers. Primary outcomes include ART initiation, retention, and TB treatment success. Secondary outcomes include time to ART initiation, adherence, change in CD4+ count, sputum smear conversion, cost-effectiveness, and acceptability. Follow-up and data abstraction are complete.

**Discussion:**

The START Study evaluates a CIP targeting barriers to early ART implementation among TB/HIV patients. If the CIP is found effective and acceptable, this study has the potential to inform care for TB/HIV patients in high-burden, resource-limited countries in sub-Saharan Africa.

## Introduction

Despite the global scale-up of antiretroviral therapy (ART), tuberculosis (TB) remains a significant cause of morbidity and mortality among people living with HIV (PLHIV). In 2014, approximately 1.2 million new TB cases globally were HIV co-infected; 74% were in Africa (1). TB is responsible for one quarter of deaths among PLHIV (1). TB case fatality rates are 16–35% in PLHIV in the absence of ART, compared with 4–9% in HIV-negative individuals (2). ART substantially reduces mortality risk by 64–95%, and is associated with a reduction in recurrent TB (3–5). Three clinical trials demonstrated that initiating ART early during TB treatment (within 2–4 weeks) greatly increases AIDS-free survival by 34–68% among individuals with advanced HIV disease (6–8), and World Health Organization (WHO) guidelines recommend ART initiation for all PLHIV as soon as possible after TB treatment initiation, regardless of CD4+ count (9).

Despite this compelling scientific evidence and strong endorsement by the WHO, data from high-burden, resource-limited countries suggest that when ART is provided to HIV-infected TB patients in programmatic settings, uptake is suboptimal, and few co-infected patients initiate ART within the recommended timeframe (10–15). Furthermore, retention in ART programs has been limited (16).

Although studies have described various interventions to address this evidence-to-program gap (10, 17, 18), few have rigorously assessed a combination approach that includes programmatic, structural, and psychosocial interventions that target the various barriers commonly described in the literature (15, 17, 19–23). In addition, there is a need for implementation science research that not only evaluates the effectiveness and cost-effectiveness of combination approaches, but also provides information on the acceptability of the interventions from both the patient and provider perspectives, as well as pragmatic information on the implementation process (24).

Lesotho is a landlocked country surrounded by South Africa with the world's highest TB incidence (852 per 100,000) (1) and the second highest HIV prevalence (23%) (25); 72% of TB patients are co-infected with HIV (1). The Government of Lesotho has made admirable strides in addressing the dual epidemics. At the time of study start in 2012, 88% of TB patients had a known HIV status and 97% of those diagnosed with HIV were placed on cotrimoxazole prophylaxis; however, the rates of ART initiation and retention during TB treatment remained suboptimal (26).

We describe the design of the Start TB Patients on ART and Retain on Treatment (START) Study, a mixed-methods, cluster-randomized implementation science study that aims to evaluate the effectiveness, cost-effectiveness, and acceptability of a combination intervention package (CIP) designed to improve early ART initiation and retention during TB treatment, as well as TB treatment success, among HIV-infected TB patients in Lesotho.

## Methods/design

The START Study is a two-arm, mixed-methods, cluster-randomized trial. Twelve health facilities with integrated TB/HIV clinics (the clusters) were randomized to deliver a CIP or standard of care (SOC), following stratification by facility type (hospital or health center).

### Study setting

The study was conducted in Berea District of the Kingdom of Lesotho, a lower middle income sub-Saharan African country of 2.1 million people (27). In 2014, 9,856 cases of all forms of TB were reported. The treatment success rate for new and relapse TB cases registered in 2013 was 70%, short of the WHO's 85% target (1).

The majority of people in Lesotho receive health care at health centers staffed by nurses, who provide outpatient care and preventive services. Complicated cases are referred to district hospitals. Nurses can initiate TB treatment for smear positive cases at the health center level; smear negative cases are referred to the hospital for evaluation and treatment initiation by a medical officer. At the health center level, TB/HIV services including provider-initiated HIV testing and counseling and the provision of cotrimoxazole prophylaxis and ART are well integrated with TB treatment and are provided as one-stop services. At the hospital level, ART is integrated within TB clinics, as per national guidelines. Typically, each clinic providing TB/HIV services is staffed by one to three nurses, plus one or two lay counselors, supported by 20–30 village health workers (VHW). TB treatment, ART, and cotrimoxazole are dispensed on-site. Sputum smear microscopy is available through specimen transport to district hospitals by motorbike on a weekly basis.

### Policy context and collaboration with the Government of Lesotho

The Ministry of Health (MOH) National Tuberculosis Program was engaged from the study conception and design stage, when applying for funding. This participatory approach (28) was adopted to ensure that the study objectives were aligned with MOH priorities for controlling the TB/HIV epidemic. The MOH is represented among the investigative team as well as the Stakeholder Advisory Committee. By engaging the MOH and other key stakeholders early and often in the planning and implementation of the study, we aimed to foster country ownership and maximize the impact of the study's findings on policy and programming.

### Facility selection

Twelve of 17 public health facilities in Berea District were selected for participation. The remaining five health facilities were excluded from the sampling frame because patient volume was very low (on average, fewer than six TB patients notified per quarter). Both hospitals (*N*=2) and health centers (*N*=10) were included to enhance generalizability. A single district was chosen in collaboration with MOH to enhance internal validity by ensuring comparability of health facilities randomized to either study arm, and maximize cost efficiency.

### Assignment to study arm

Assignment to a study arm was done at the health facility level as opposed to the individual participant level. Health facilities were stratified by facility type (i.e. hospital or health center), and then numbered sequentially within each stratum. Intervention status was randomly assigned within each stratum by an investigator using SAS v. 9.3 (SAS Institute, Cary, NC). All HIV-infected TB patients at facilities assigned to the SOC condition received standard of care supported by the Lesotho MOH, while all those enrolled in facilities assigned to the CIP condition received the standard of care plus the intervention package. Given the nature of the interventions, participants, health care workers, and study staff were not blinded to the assigned study arm.

### Standard of care

At health facilities randomly assigned to SOC, the usual procedures for the management of HIV-infected TB patients were followed. Lay counselors offered HIV testing to all TB patients, and integrated treatment for TB and HIV is provided by nurses who are trained in the National Guidelines for Tuberculosis (29). As per national guidelines, HIV-infected TB patients are to be started on ART 2–4 weeks after initiating TB treatment, regardless of CD4+ count. There is no standardized, nationally approved curriculum to provide patients with treatment and adherence literacy.

Directly observed therapy for TB may be provided by a nurse at the health facility, but is generally provided by a treatment supporter, who is usually a family member. Designation of a treatment supporter by the patient is not verified by the nurse. Treatment supporters are not required to accompany patients to clinic visits, and thus often do not receive any education on TB or adherence. VHW are trained to be treatment supporters; however, there is no formal referral system to ensure that patients are linked with them. VHW are minimally supervised, and they communicate infrequently with providers at nearby health facilities.

TB patients return to the health facility monthly for a 30-day supply of medications and monitoring of side effects and adherence. The tracing of patients lost to follow-up is usually attempted on a quarterly basis but outreach activities are often limited owing to lack of transportation and means of communication.

### Combination intervention package

At health facilities randomly assigned to CIP, the intervention was delivered to all TB/HIV patients during regular clinic visits as part of routine care. All nurses and VHW affiliated with the TB/HIV clinic were trained to implement the CIP. CIP contained programmatic, structural, and psychosocial components, including: 1) nurse training and mentorship in TB/HIV co-treatment, using a clinical algorithm; 2) reimbursement of transportation costs to monthly clinic visits for patients and treatment supporters; 3) health education for patients and treatment supporters by VHW using a treatment literacy curriculum; and 4) real-time adherence support using short message service (SMS) text messaging and trained VHW. Table 1 shows a comparison of the study arms.

**Table 1:** Comparison of study arms

	Standard of care (SOC)	Combination intervention package (CIP)
Training on national TB guidelines for nurses	X	X
ART provision to TB/HIV patients in integrated clinics	X	X
Patient-identified treatment supporter for TB treatment	X	X
Nurse training and mentorship in TB/HIV co-treatment using a clinical algorithm		X
Reimbursement of transportation costs to monthly clinic visits for patients and treatment supporters		X
Health education for patients and treatment supporters by village health workers using TB/HIV treatment literacy curriculum		X
Real-time adherence support using automated SMS text messages and trained village health workers		X

#### Programmatic components of CIP: nurse training and mentorship in TB/HIV co-treatment, using a clinical algorithm

All nurses working in CIP clinics participated in a 2-day training on the scientific evidence and guidelines regarding treatment of TB/HIV co-infection, the importance of early ART initiation during TB treatment to improve survival, and use of a clinical algorithm to manage TB/HIV patients. Job aids, such as laminated desk charts and posters depicting the algorithm, were provided for each consultation room to support intervention delivery. Clinic nurses used the algorithm to initiate ART; monitor for adherence, treatment response, and side effects; and diagnose and manage adverse events, including immune reconstitution inflammatory syndrome (IRIS). Nurses explained to TB/HIV patients that ART can improve survival, encouraged ART initiation, and referred patients to the facility-based lead village health worker (LVHW) for further education. Nurses also delivered specific adherence messages and assessed ART and TB treatment adherence at follow-up visits, referring patients to the LVHW for additional adherence counseling. A nurse program monitor visited the clinics two to three times per month to provide ongoing mentorship in TB/HIV co-treatment to the clinic nurses and LVHW and provided refresher training as needed.

#### Structural component of CIP: reimbursement of transportation costs

TB/HIV patients initiating ART were asked to bring their treatment supporter to their monthly clinic visits, so that they could participate in education and counseling sessions with the LVHW, and discuss any adherence problems. Both patients and treatment supporters were reimbursed for transportation costs in the amount of 15 loti (approximately 1.4 USD) after each clinic visit.

#### Psychosocial components of CIP

Psychosocial components of CIP were delivered by VHW, whose role encompassed social support, system navigation, referrals, and advocacy. VHW responsibilities included providing information; communicating with patients; encouraging ART initiation, adherence to ART and TB treatment, and retention in care; offering support and empathy; providing referrals; and advocating for patients. In order to improve VHW oversight and coordination, one to two VHW were assigned to be LVHW at each facility, according to patient volume. LVHW were chosen by a joint committee of their peers and the nurse in charge, and received a monthly stipend of 750 loti (approximately 70.0 USD). All VHW completed a 4-day training in both didactic learning and interactive techniques, including case studies and role-playing to develop skills. LVHW completed an additional 2 days of training on their additional responsibilities at the health facility, as well as their role supervising VHW in the community.

LVHW assigned new TB/HIV patients initiating ART to a community-based VHW, who provided adherence support between clinic visits. VHW conducted home visits and contacted patients by phone, weekly for the duration of TB treatment, to discuss adherence and side effects. They also followed up with patients who missed clinic appointments, contacting the LVHW or nurse for guidance when necessary, and linked patients with community-based services as needed. VHW were provided with airtime vouchers (40 loti, approximately 3.7 USD) each month.

LVHW used a TB/HIV treatment literacy curriculum to provide group health education sessions at the health facility. They also met individually with TB/HIV patients and their treatment supporters to provide ART preparation and adherence counseling for the duration of their TB treatment. A scripted, illustrated flipchart was developed for LVHW. It provides information on the natural history of HIV, importance of early ART initiation, use of TB and antiretroviral medications, common side effects, and the importance of adherence. In response to an expressed need from LVHW, a second flipchart that provides information on and support for HIV disclosure was also developed.

In addition, LVHW enrolled TB/HIV patients initiating ART and their treatment supporters in an automated text messaging system supported by Dimagi Commcare (www.dimagi.com/), using a customized mobile phone application. This system sent text messages in Sesotho, including medication adherence messages and appointment reminders. Adherence messages used code words (Did you eat your meal today?) to protect patients’ confidentiality. They were sent on a daily basis during the first month of ART, then on a patient-specified schedule (daily or weekly) for the remainder of TB treatment. Appointment reminders were sent 2 and 1 day prior to each clinic visit. The time of day for all messages was chosen by the recipient. LVHW described text messaging in detail and practiced receiving text messages with patients and treatment supporters until they felt comfortable with the technology. LVHW also dispensed airtime vouchers (40 loti, approximately 3.7 USD) to patients and treatment supporters at their monthly clinic visits, so that they were able to communicate with their VHW or nurse in case of difficulties.

In addition, LVHW managed the clinic appointment system, including scheduling appointments, checking the appointment book to confirm attendance, and notifying the assigned VHW if any patients missed their appointments. They escorted patients to other services at the health facility when needed. They also supervised the VHW in the community on a weekly basis using a standardized checklist, and held monthly meetings with the VHW and clinic nurses at the health facility to discuss patient challenges.

### Study participants

**Fig. 1:**
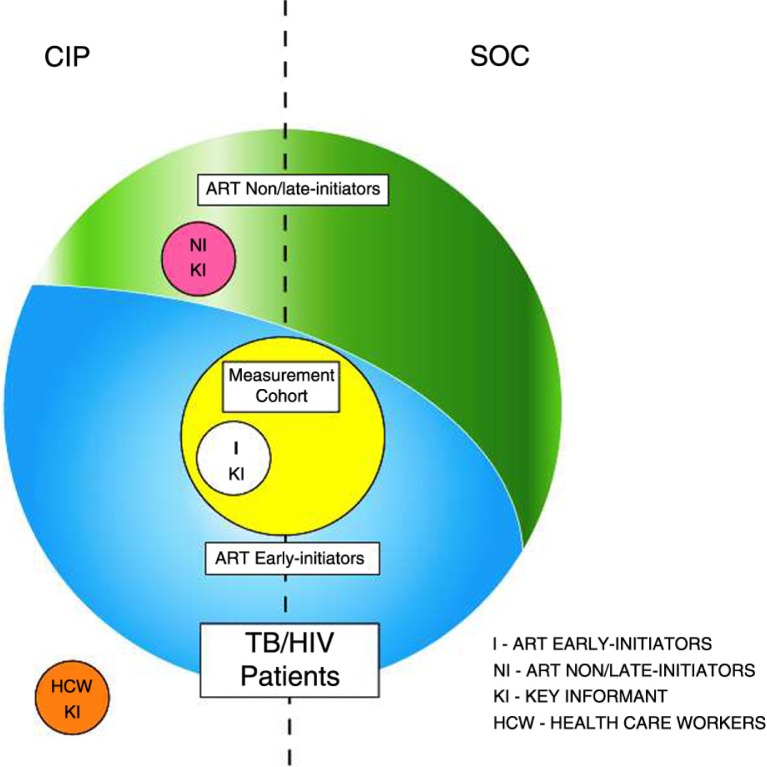
Study Participants. *All newly registered TB/HIV patients* at study sites in both conditions are represented by the large circle; those who initiated ART during the first 2 months of TB treatment (ART early initiators) are depicted in blue, while those who did not initiate ART or initiated ART after the first 2 months of TB treatment (ART non/late initiators) are depicted in green. A sample of ART early initiators from study sites in both conditions who enrolled in the *measurement cohort* are represented by the yellow circle. Key informants at CIP study sites included: 1) *ART early initiators (I KI)*, depicted by the white circle; 2) *ART non/late initiators (NI KI)*, depicted by the pink circle; and 3) health care workers *(HCW KI)*, depicted by the orange circle.

The different groups of study participants are shown in Fig. 1.

#### All newly registered TB/HIV patients

Routinely collected data were abstracted for all TB/HIV patients newly registered for TB treatment at the 12 study clinics during the first 24 months of observation.

#### Measurement cohort

In addition, a measurement cohort of TB/HIV patients initiating ART was recruited. Inclusion criteria were 1) HIV-infected; 2) on TB treatment; 3) initiated ART within 2 months of TB treatment initiation; 4) aged 18 or older; 5) English- or Sesotho-speaking; and 6) capable of and willing to provide informed consent within 3 working days of ART initiation. Patients with multidrug-resistant TB were excluded from the measurement cohort.

#### Key informants

Three groups of key informants were recruited from CIP clinics: 1) ART early initiators; 2) ART non/late initiators; and 3) health care workers.

The inclusion criteria for ART early initiators were 1) measurement cohort participation at a CIP clinic; and 2) ART initiation at minimum 2 weeks prior to completion of key informant interview.

Inclusion criteria for ART non/late initiators were 1) HIV-infected; 2) recently completed TB treatment at CIP clinic (if non-initiator) or on TB treatment for 2 months or longer (if late initiator); 3) ART-naïve or ART initiated 2 months or longer after TB treatment initiation; 4) aged 18 or older; 5) English- or Sesotho-speaking; and 6) capable of and willing to provide informed consent. Patients with multidrug-resistant TB were excluded. Initially, we aimed to recruit ART non-initiators only; however, inclusion criteria were broadened to include late initiators in month 7 of key informant recruitment, as sufficient numbers of TB/HIV patients who were ART-naïve could not be identified.

Inclusion criteria for health care workers were 1) nurse or VHW working in a CIP clinic or VHW working in the community and affiliated with CIP clinic; 2) aged 18 or older; 3) English- or Sesotho-speaking; and 4) capable of and willing to provide informed consent. Nurses and VHW working in or affiliated with a SOC clinic were excluded.

### Recruitment

#### Measurement cohort

Nurses were asked to inform TB/HIV patients initiating ART about the study and to refer them to the research assistant (RA) if they were interested in obtaining more information. To achieve steady enrollment across study arms, a monthly enrollment quota was set for each site and, consecutively, eligible consenting patients were enrolled until the target number of participants was reached.

#### Key informants

ART early initiators were recruited from the measurement cohort, so that monthly questionnaire data could be used to characterize them. Heterogeneous purposive sampling was utilized (30, 31), according to the following recruitment targets: 1) equal numbers of men and women; 2) equal numbers of participants aged 18–35 years and older; and 3) a proportional number of participants at each CIP clinic, based on patient intake. ART non/late initiators were referred to the RA by clinic nurses. Convenience sampling was utilized (30), according to the following recruitment targets: 1) equal numbers of men and women; and 2) a proportional number of participants at each CIP clinic, based on patient intake. Health care workers were referred to the RA by the nurse in charge. Convenience sampling was utilized, with the following recruitment targets: 1) at least one nurse per clinic; 2) at least one LVHW per clinic; and 3) at least one community-based VHW per clinic.

For all participant groups, RA met with potential participants in a private area to provide further information about the study using a standardized script, assess eligibility, and obtain written informed consent.

### Data collection

#### All newly registered TB/HIV patients

RA abstracted demographic, clinical, and laboratory data from the clinic TB and ART registers and patient TB treatment and HIV care cards using a standardized tool. Standardized data quality assurance procedures were implemented at all study sites to mitigate the amount of missing data abstracted from clinic records. Patient information was recorded in disparate paper and electronic systems that were not always incorporated into the patient files. Study staff conducted regular reviews of patient files, various clinic registers, laboratory information systems, and other source documents to ensure a high standard of completeness of the key study variables.

#### Measurement cohort

Consenting participants in both conditions completed the same standardized assessments, including interviewer-administered questionnaires at baseline and monthly follow-up visits coinciding with their clinic visits and monthly phone-based unannounced pill counts, for the duration of TB treatment (6–9 months). All interviews were conducted in either Sesotho or English, based on participant preference. In addition, RA abstracted detailed information from clinic records on participants’ clinic visits and medication refills.

Interviewer-administered questionnaires

The baseline questionnaire included measures of sociodemographic characteristics (32), patient costs, disclosure of HIV status, HIV- and TB-related knowledge and attitudes, depression (PHQ-9) (33), alcohol dependence (AUDIT) (34), barriers to medical care, social support, health literacy, social desirability (35), and side effects. The follow-up questionnaire included measures of self-reported 30-day adherence (36), side effects, and patient costs. The end-of-treatment questionnaire included the same measures included in the baseline and follow-up questionnaires, as well as measures of utilization and acceptability of services.

Unannounced pill counts

Participants were called on their mobile phone at an undisclosed time between their monthly study visits and asked to count the pills remaining in their pill bottles or plastic envelopes (37).

#### Key informants

In-depth qualitative interviews were conducted with consenting key informants using semi-structured interview guides (38) that were tailored for each key informant group. Interviews were audio recorded, transcribed verbatim, and translated from Sesotho to English. Acceptability, preferences, and utilization of intervention components were explored among ART initiators. Reasons for the lack of or late initiation of ART were explored among ART non/late initiators. Experiences delivering the intervention, perceived barriers and facilitators, perceptions about acceptability, and the ease of uptake and delivery of the intervention components were explored among health care workers.

#### Cost drivers

RA administered cost surveys to nurses and VHW on a monthly basis to capture the program costs associated with CIP implementation.

#### Process documentation

Several instruments were also completed by study staff to document the implementation process. A brief semi-structured *Program Characteristics Survey* was administered by the RA to the nurse in charge at each clinic in both study conditions, prior to study implementation, and on a monthly basis thereafter. The survey tracked the implementation of intervention components to assess fidelity, potential contamination, as well as system-level factors that may impact CIP implementation. At each CIP site, the RA also completed an intervention receipt log for mobile airtime, SMS messages, and transportation reimbursement to document the dosage of intervention components received by each patient, and the program monitor completed a supervision checklist during each mentorship visit to assess intervention quality (39).

Copies of data collection instruments are available from the corresponding author upon request.

### Data management

Data for participant interviews and chart abstractions were collected on paper-based forms. Completed forms were stored in a locked filing cabinet at each clinic site, and on a weekly basis were transported in a locked box to a field study office for entry in the study database. The database was encrypted, password-protected, and contained established quality control measures, including skip patterns, range limitations, and consistency checks to enhance the accuracy and completeness of the data collected. The database was backed up nightly to an encrypted external hard drive maintained in a locked filing cabinet.

Throughout the study, four levels of review were conducted to ensure the completeness and accuracy of the study data. First, the study coordinator conducted random checks of 10% of the completed study forms for errors and completeness. Second, the data manager conducted a random verification of 10% of the records newly entered into the study database against the paper data collection forms. Third, during external monitoring visits, the project coordinator reviewed 10% of the data collection forms entered into the study database for completeness and accuracy, comparing data collection forms with source documentation and hardcopy forms with the study database. In addition, the data analyst ran code to check for missing data and inconsistencies and flagged problematic observations for the study team to verify against paper-based study forms or with source documentation at the study site.

### Primary and secondary endpoints

Primary outcomes include: 1) ART initiation, 2) ART retention, and 3) TB treatment success. Secondary outcomes include: 1) time to ART initiation, 2) ART adherence, 3) change in CD4+ count, 4) sputum smear conversion, 5) TB treatment adherence, 6) cost-effectiveness, and 7) acceptability. All outcomes pertain to the individual participant level. ART initiation is defined as the percentage of TB/HIV patients newly registered during the period of observation who initiated ART during TB treatment, based on review of clinic records. ART retention is defined as the percentage of measurement cohort participants who attended their 6-month clinic visit (within a 1-month window) and reported ART use. Deaths and transfers will be considered not retained. TB treatment success is defined as the percentage of TB/HIV patients whose TB treatment outcome is documented in the TB register or treatment card as treatment completion or cure, as defined by the WHO (40). Time to ART initiation will be expressed as the number of days from TB treatment initiation to ART initiation. Adherence is determined separately for ART and TB treatment and defined by the percentage of total prescribed doses ingested, averaged across medicines for each month of treatment, from the unannounced pill counts. Change in CD4+ count is defined as the difference in CD4+ count 6 months after TB treatment initiation. Sputum smear conversion is defined as the percentage of smear positive pulmonary TB cases that converted to smear negative after 8 weeks of treatment (40). Incremental cost-effectiveness will be estimated as the ratio of the incremental costs of the CIP per ART initiation, retention, and TB treatment success. Acceptability of intervention components will be characterized via in-depth qualitative analysis and interpretation (41). Table 2 lists the study outcomes for each group of participants.

**Table 2:** Study outcomes

Study outcome	All TB/HIV patients	Measurement cohort	Key informants – ART early initiators	Key informants – ART non/late initiators	Key informants-health care workers
ART initiation^a^	X				
ART retention^a^		X			
TB treatment success^a^	X				
Time to ART initiation	X				
ART adherence		X			
Change in CD4+ count		X			
TB treatment adherence		X			
Sputum smear conversion	X				
Incremental cost-effectiveness		X			
Acceptability of intervention components			X	X	X
Reasons for ART non/late initiation				X	

a Primary outcomes.

### Sample size and power calculations

Power calculations for all TB/HIV patients were based on our primary outcomes ART initiation and TB treatment success. Based on previous programmatic data, we anticipated an average of 100 newly registered TB/HIV patients at each of the 12 study clinics over the first 24 months of observation. When the study was designed, available data demonstrated that only 27% of HIV-infected TB patients in Lesotho had received ART, and the TB treatment success rate was 70% for new smear positive cases, 64% for new smear negative and extrapulmonary cases, and 62% for retreatment cases (HIV-positive and HIV-negative cases combined) (42). Assuming a two-sided Farrington & Manning Likelihood Score Test with *α*=0.05 and an intra-cluster correlation coefficient (ICC) of 0.05, we will have 83% power to detect a difference in ART initiation from 40% (SOC) to 60% (CIP), and 91% power to detect a difference in TB treatment success from 65% (SOC) to 85% (CIP) (PASS 2008, NCSS Statistic Software). As per standard practice, patients who die or are lost to follow-up before ART initiation or TB treatment completion are considered to have not initiated ART or to have not achieved TB treatment success, respectively, and thus power calculations for these outcomes do not include an allowance for attrition.

Power calculations for measurement cohort participants were based on our primary outcome ART retention. Assuming 100 TB/HIV patients in each of the 12 study clinics, 40% ART initiation at SOC clinics, and at least 80% study enrollment, we aimed to enroll 384 participants (32 per clinic) initiating ART in the measurement cohort. Assuming a two-sided Farrington & Manning Likelihood Score Test with *α*=0.05 and ICC of 0.05, we will have 87% power to detect a difference in ART retention from 70% (SOC) to 90% (CIP).

### Data analysis

An intent-to-treat analysis will be used. In addition, when measuring adherence in the measurement cohort, an as-treated analysis will be used to exclude participants who died or discontinued medications due to adverse events. Generalized linear mixed models (Proc Glimmix procedures in SAS v9.3 with a random intercept for study site) will be applied to test for a difference between study arms for dichotomous (ART initiation, ART retention, TB treatment success, sputum smear conversion) and continuous (ART adherence, change in CD4+ count, TB treatment adherence) outcomes; frailty models (Proc Phreg procedures in SAS v9.3 with random intercept for study site) will be utilized for time to event analysis (time to ART initiation) to account for the clustering of characteristics that may occur among patients attending the same study site. These approaches will provide appropriate adjustments to the standard errors accounting for potential non-independence of observations (43). Models will include fixed effects for study condition, patient characteristics, time-varying adherence measures and CD4+ count (for change in CD4+ count) and random effects for site. Participants who transferred to another site before ART initiation will be censored, and death before ART initiation will be treated as a competing risk.

For the cost-effectiveness analysis, a model will be created to estimate the incremental cost of CIP relative to SOC. The costs will be composed primarily of the variable costs involved in training nurses and convening VHW, but will also include the costs of administering SMS messages and the fixed costs involved in administering the intervention. The incremental cost-effectiveness of CIP will be estimated as the ratio of the incremental costs to incremental effectiveness with effectiveness measured in terms of the study's primary outcomes.

For analysis of acceptability of the CIP, two investigators will independently review the first five interviews from each group of key informants to develop a preliminary checklist of codes using a grounded theory framework (41, 44). The individual checklists will be compared and reconciled to create a coding dictionary, which will be used by the study team to independently code each interview. Additional codes will be added if they emerge in the analysis of subsequent interviews. Codes will accordingly serve as building blocks for qualitative concepts and themes. Themes will be cross-checked to enhance inter-rater reliability, and negative case analysis and critical reflexivity will be used to strengthen analytic credibility (41, 45). Data from patients and providers will be analyzed separately and comparatively. Data analysis will explore contextual factors related to the acceptability of CIP components among patients; detect differences, if any, between men and women; and will shed light on common and divergent characteristics of patients’ and providers’ barriers and facilitators.

### Monitoring

As this is an implementation science study with minimal risks, a data monitoring committee was not needed. Study staff was trained to assess for adverse events and notify the principal investigator immediately if they learned of an adverse event. If there was an adverse event, an incident report was to be completed describing the incident, what caused it, and steps that would be taken to prevent recurrence, and the Columbia University Medical Center Institutional Review Board and the Lesotho National Health Research and Ethics Committee were to be informed according to their respective reporting guidelines.

Internal monitoring of each study site was performed by the study coordinator on a monthly basis, to ensure that each site was adhering to the study protocol and standard operating procedures. External monitoring visits were performed semi-annually by a US-based project coordinator and included review of each site's performance, the completion and storage of data collection forms, data entry, and adherence to confidentiality guidelines.

### Ethics and consent process

The protocol and any modifications were reviewed and approved by the Columbia University Medical Center Institutional Review Board and the Lesotho National Health Research and Ethics Committee. RA obtained written informed consent from all measurement cohort participants and key informants completing in-depth interviews.

Consent forms and all of the identifying information obtained at study enrollment to track participants were stored in separate locked filing cabinets at study sites in a locked room. Upon enrollment, each participant was assigned a unique identification number. The study questionnaires, data abstraction forms, and databases included participant unique identification numbers only; no participant names or identifiers were recorded. A master list with each participant's name and unique identification number is stored in a locked cabinet, and will be maintained only long enough to permit the investigators to review and audit the data, following which this document will be destroyed. Investigators have and will maintain access to the full trial dataset.

### Dissemination

A dissemination strategy was developed to ensure that study findings are shared with key stakeholders, regardless of the magnitude or direction of effect. This strategy includes a dissemination meeting in Lesotho with the MOH, District Health Management Team, health care workers, and patients from participating health facilities; a dissemination report for the MOH; presentations at scientific conferences; and publications in peer-reviewed journals. Investigators will adhere to recommendations from the International Committee of Medical Journal Editors regarding authorship. Data, from which all identifiers have been removed, will be made publicly available following the publication of primary and secondary outcome papers in accordance with Open Data Policy of the United States Agency for International Development (USAID) (46).

### Trial status

The study commenced with the enrollment of measurement cohort participants in April 2013 and the recruitment of key informants in March 2014; the enrollment of all participants was completed in March 2015 and follow-up procedures including clinical and laboratory record abstraction was completed in March 2016.

## Discussion

The START Study aims to evaluate a CIP designed to improve early ART initiation and retention during TB treatment, as well as TB treatment success, among HIV-infected TB patients in Lesotho. The study utilizes an implementation science framework to not only assess the effectiveness and cost-effectiveness of the combination approach, but also provide information on the acceptability of the interventions from both the patient and provider perspectives, as well as pragmatic information on the implementation process. The study interventions target known programmatic, structural, and psychosocial barriers to early ART implementation in high-burden, resource-limited settings, and were selected for their promise, practicality, and feasibility of implementation and scale-up in TB/HIV programs in diverse contexts. The goal of this study is to improve health outcomes among PLHIV in Lesotho, and generate generalizable knowledge that could be applied to similar high-burden, resource-limited settings.

We used a cluster-randomized design for a number of reasons. First, the delivery of study interventions at the health facility level is conducive to the study's implementation science approach as it mimics the way in which the CIP would be delivered outside of a study setting. In addition, it was considered more feasible for the clinic staff to provide all patients at the site with the same intervention package, rather than deliver the CIP to some patients and not others, which would be the case in an individual-randomized design. Furthermore, though there were no data to support that the CIP would be superior to the SOC, individual randomization might have resulted in some patients randomized to SOC feeling that they were receiving substandard care, which in turn might have influenced their engagement in care and, consequently, the key study outcomes of ART initiation and retention.

Our study has several strengths. First, the cluster-randomized approach permits the causal attribution of observed outcomes to the CIP by comparing them to the counterfactual scenario, while reducing selection bias (47). Second, the use of mixed methods (48, 49), whereby the collection and analysis of quantitative data is followed by the utilization of qualitative research tools in a sequential explanatory phase, will allow us to evaluate the acceptability and utilization of intervention components, and collect reasons for the lack of or late initiation of ART in TB/HIV patients. Third, the participatory approach, with meaningful stakeholder engagement in both the design and implementation phases, has fostered MOH ownership at the national and district levels, and will help to ensure the successful integration of study findings in policy and programmatic contexts. In addition, we are testing an innovative, multifaceted intervention that builds on the evidence base of prior scientific work, while addressing the diverse barriers to ART initiation and retention in TB/HIV patients. Finally, the selected study sites have adequate heterogeneity, covering facilities in both urban and rural locations in low-land, foothill, and mountainous areas, which will strengthen the external validity of findings.

The limitations of the study design include the potential for unanticipated health system inefficiencies to impact CIP implementation, including interruptions in health care worker availability, laboratory commodities, and drug supplies. However, any changes in these elements are likely to be similar across study arms and reflect system dynamics captured in implementation science. In addition, the reliance on routinely collected programmatic data for some study outcomes means that data may be incomplete for some study participants. Standardized data quality assurance procedures implemented at all study sites aim to mitigate the amount of missing data. Furthermore, as the study is limited to one district, there is the potential for contamination and migration, which would result in a decrease in power to detect a difference between study arms. However, given the goal of assessing CIP effectiveness in a health systems context, it is important to evaluate the impact in realistic programmatic scenarios, while seeking to understand the findings by monitoring spillover and crossover through process documentation. Last, the study design precludes evaluation of the effectiveness of individual components of the CIP, which would require a considerably larger sample size. However, qualitative results will highlight patient and provider perspectives on the utility of various intervention components, and process data will demonstrate utilization of each study intervention.

## Conclusions

The START Study aims to improve early ART initiation and retention during TB treatment. Achieving this goal will result in improved health outcomes among PLHIV as well as reduced HIV and TB transmission in the population. With one of the world's most severe epidemics of HIV and TB, Lesotho would benefit greatly from the identification of an effective strategy to integrate this life-saving treatment into TB service delivery programs. If this study demonstrates that the CIP is effective, cost-effective, and acceptable, these findings can be used to advocate for its adoption as standard of care for HIV-infected TB patients in other high-burden, resource-limited countries in sub-Saharan Africa.

## Supplementary Material

The START Study to evaluate the effectiveness of a combination intervention package to enhance antiretroviral therapy uptake and retention during TB treatment among TB/HIV patients in Lesotho: rationale and design of a mixed-methods, cluster-randomized trial

## Paper context

Initiating antiretroviral therapy early during tuberculosis treatment increases the survival of HIV-infected tuberculosis patients; however, implementation is suboptimal. The START Study is a mixed-methods cluster-randomized trial aimed at evaluating the effectiveness, cost-effectiveness, and acceptability of a combination intervention package to improve early antiretroviral therapy initiation, retention, and tuberculosis treatment success among HIV-infected tuberculosis patients in Lesotho. This study has the potential to inform care for HIV-infected tuberculosis patients in high-burden, resource-limited countries in sub-Saharan Africa.
