# Supplementary material for: The START Study to evaluate the effectiveness of a combination intervention package to enhance antiretroviral therapy uptake and retention during TB treatment among TB/HIV patients in Lesotho: rationale and design of a mixed-methods, cluster-randomized trial
Source: Glob Health Action. 2016 Jun 27;9:10.3402/gha.v9.31543. doi: 10.3402/gha.v9.31543 (PMC4926099; doi:10.3402/gha.v9.31543)
Supplement: The START Study to evaluate the effectiveness of a combination intervention package to enhance antiretroviral therapy uptake and retention during TB treatment among TB/HIV patients in Lesotho: rationale and design of a mixed-methods, cluster-randomized trial [file GHA-9-31543-s001.doc]

Supplemental Figure. Schedule of study interventions, measurement cohort enrollment, and assessments.

|  | **STUDY PERIOD** | | | | | | | |
| --- | --- | --- | --- | --- | --- | --- | --- | --- |
|  | **Allocation*** | **Enrollment** | **Post-enrollment**** | | | | | **End of TB tx**** |
| **TIMEPOINT** |  | **0** | ***Month 1*** | ***Month***  ***2*** | ***Month 3*** | ***Month 4*** | ***Month 5*** | ***Month***  ***6*** |
| **INTERVENTIONS:** |  |  |  |  |  |  |  |  |
| ***Combination Intervention Package*** |  |  |  |  |  |  |  |  |
| ***Standard of Care*** |  |  |  |  |  |  |  |  |
| **ENROLLMENT:** |  |  |  |  |  |  |  |  |
| **Eligibility screen** |  | X |  |  |  |  |  |  |
| **Informed consent** |  | X |  |  |  |  |  |  |
| **ASSESSMENTS:** |  |  |  |  |  |  |  |  |
| ***Baseline interview*** |  | X |  |  |  |  |  |  |
| ***Follow-up interview*** |  |  | X | X | X | X | X |  |
| ***End-of-treatment interview*** |  |  |  |  |  |  |  | X |
| ***Unannounced pill count****** |  |  | X | X | X | X | X | X |
| ***Data abstraction form*** |  |  |  |  |  |  |  | X |

*As this is a cluster-randomized trial, allocation occurred at the cluster (health facility) level.

**Participants who received more than 6 months of TB treatment had up to 8 monthly follow-up visits and an end-of-treatment visit that occurred up to month 9.

***Unannounced pill counts occurred prior to the monthly follow-up interviews and end-of-treatment interview.
